# Inhibition of cyclic diadenylate cyclase, DisA, by polyphenols

**DOI:** 10.1038/srep25445

**Published:** 2016-05-06

**Authors:** Clement Opoku-Temeng, Herman O. Sintim

**Affiliations:** 1 Department of Chemistry, Purdue University, West Lafayette, IN 47907, USA; 2 Center for Drug Discovery, Purdue University, West Lafayette, IN 47907, USA; 3 Graduate program in Biochemistry, University of Maryland, College Park, Maryland 20742, USA

## Abstract

Cyclic di-AMP has emerged as an important signaling molecule that controls a myriad of functions, including cell wall homeostasis in different bacteria. Polyphenols display various biological activities and tea polyphenols in particular have been shown to possess among other properties antioxidant and antibacterial activities. Certain tea polyphenols, such as catechin and epigallocatechin gallate, have been used to augment the action of traditional antibiotics that target the cell wall. Considering the expanding role played by cyclic dinucleotides in bacteria, we investigated whether the action of polyphenols on bacteria could be due in part to modulation of c-di-AMP signaling. Out of 14 tested polyphenols, tannic acid (TA), theaflavin-3′-gallate (TF2B) and theaflavin-3,3′-digallate (TF3) exhibited inhibitory effects on *B. subtilis* c-di-AMP synthase, DisA. TF2B and TF3 specifically inhibited DisA but not YybT (a PDE) whilst TA was more promiscuous and inhibited both DisA and YybT.

Nucleotides play critical roles in cells, some of which include serving as a source of energy, as components of biomolecules like DNA and RNA and as cofactors of enzymes. It has long been known that mononucleotides such as cAMP and ppGpp regulate several processes in bacteria^1^,^2^. In the late 1980s Benziman and colleagues identified cyclic dinucleotide bis-(3′ -5′ )-cyclic dimeric guanosine monophosphate (c-di-GMP) as an allosteric regulator in the bacterium *Acetobacter xylinum* (now called *Gluconacetobacter xylinus*)^3^. It would take close to two decades before the microbiology community fully appreciated that c-di-GMP is a master regulator of bacterial physiology^1^. In Gram-negative bacteria c-di-GMP controls the transition from planktonic to the biofilm state and there has been an explosion of research activities dedicated to unravelling the intricacies of c-di-GMP signaling. Just as c-di-GMP research was taking shape, another related cyclic dinucleotide, bis-(3′ -5′ )-cyclic dimeric adenosine monophosphate (c-di-AMP) was identified by Hopfner and colleagues during their study of the *Bacillus subtilis* checkpoint protein, DNA integrity scanning protein A (DisA)^4^.

Just like the analogous c-di-GMP, c-di-AMP is also emerging as an important signaling second messenger in several bacteria and has been found to regulating several physiological processes including but not limited to cell wall homeostasis^5^,^6^, fatty acid metabolism^7^, cell size regulation^8^ and virulence^5^ (Fig. 1). C-di-AMP has been found to be mainly produced predominantly in Gram-positive Firmicutes, Actinomycetes and mycobacteria^2^,^9^. The intracellular levels of c-di-AMP are tightly regulated by two opposing enzymes: diadenylate cyclases (DAC), which synthesize c-di-AMP from two molecules of ATP/ADP and phosphodiesterases (PDE), which degrade c-di-AMP into pApA or AMP^10^,^11^,^12^,^13^. This tight regulation is important in keeping an optimal intracellular c-di-AMP concentration as overproduction or underproduction of the signaling molecule has been observed to cause interesting changes in bacteria physiology^5^,^8^,^14^. In *Listeria monocytogenes*, overexpression of PdeA led to low levels of intracellular c-di-AMP and resulted in reduced growth rate and avirulent phenotype^5^. Decreased intracellular concentration of c-di-AMP in *L. monocytogenes* also resulted in a higher susceptibility to peptidoglycan-targeting antibiotics^5^. An opposite observation was made when the PDE GdpP of *Staphylococcus aureus* was deleted, leading to an increase in peptidoglycan cross-linking and resistance to cell wall-targeting antibiotics^8^.

Studies that aimed to knock out the DAC gene however proved futile since the DAC domain in several bacteria, including the pathogens *L. monocytogenes*^15^ and *S. pneumoniae*^13^,^16^ is essential. Thus to study the role of DAC in such bacteria, researchers have resorted to conditional depletion of the gene^5^. Arguably, there is a need to identify small molecules that can modulate the functions of c-di-AMP metabolism enzymes for use as chemical probes to interrogate c-di-AMP-mediated processes. Also, since c-di-AMP has been shown to affect peptidoglycan synthesis or remodeling, it is likely that inhibitors of c-di-AMP synthesis or degradation could also be utilized as antibacterial agents or used in synergy with traditional antibiotics.

We have been interested in the development of technologies that could aid the identification of inhibitors of cyclic dinucleotide metabolism enzymes^17^,^18^,^19^. These probes have facilitated the identification of the first DisA inhibitor, bromophenol thiohydantoin^20^,^21^, which are weak inhibitors of DisA. In an effort to discover more potent inhibitors of DisA, and motivated by the dazzling arrays of enzymes that polyphenols regulate^22^,^23^, we turned our attention to evaluating polyphenols as DisA inhibitors. The biological properties of plant polyphenols have long been established^24^,^25^. Tea polyphenols for example are known for their antioxidant properties and have been studied for their anticancer, antiviral and antibacterial properties^26^,^27^. Strikingly, some tea polyphenols like catechin have been shown to potentiate the action of antibiotics, particularly cell wall targeting antibiotics like oxacillin and ampicillin, against methicillin resistant *S. aureus*^28^. We were therefore interested in investigating if polyphenols could inhibit c-di-AMP metabolism proteins. Since c-di-AMP has emerged as important second messenger that regulates diverse processes in bacteria, we wondered if perhaps some of the activity of polyphenols against bacteria was due to inhibition of c-di-AMP metabolism enzymes. Thus we tested 14 polyphenols (Fig. 2b and Supplementary Fig. S1) against *B. subtilis* DisA.

## Results

### Coralyne assay identifies TA, TF2B and TF as DisA inhibitors

**Figure 2:**
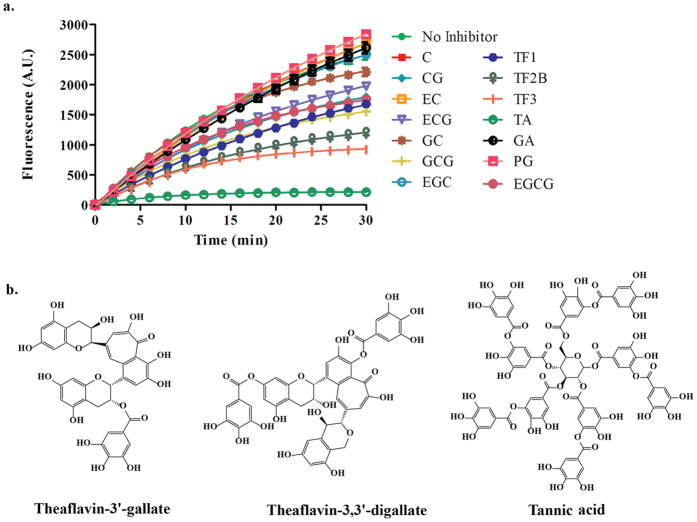
Screening of polyphenols against DisA. (**a**) Coralyne assay results of 14 polyphenols screened against DisA (1 μ M); λ ex =  420 nm and λ em =  475 nm. Polyphenols that yielded at least 50% inhibition were selected for further analysis. (**b**) Structures of the three polyphenols that were found to inhibit DisA activity. The structures of the remaining compounds can be found in Supplementary Fig. S1.

We utilized the coralyne assay^17^ developed by our group to evaluate the inhibitory effect of 14 polyphenols [gallic acid (GA), propyl gallate (PG), (-)-catechin (C), (-)-catechin gallate (CG), (-)-epicatechin (EC), (-)-epicatechin gallate (ECG), (-)-gallocatechin (GC), (-)-gallocatechin gallate (GCG), (-)-epigallocatechin (EGC), (-)-epigallocatechin gallate (EGCG), (-)-theaflavin (TF1), (-)-theaflavin 3′ -monogallate (TF2B), (-)-theaflavin-3,3′ -digallate (TF3) and tannic acid (TA)] on DisA. For structures of these compounds, see Fig. 2b and Supplementary Fig. S1. From the coralyne assay results, we selected compounds that yielded 50% or more inhibition, after 30 min. At 20 μ M inhibitor concentration and 1 μ M DisA concentration, TA completely inhibited c-di-AMP formation (Fig. 2a). TF2B and TF3 also inhibited DisA activity, albeit not as potent as tannic acid (Fig. 2a). It appears that as the number of gallates on a polyphenol increased, so did the potency of inhibition. For example, TF1, TF2B and TF3 contain the same theaflavin moiety and only differ by the number of attached gallate units (TF1 contains no gallates; TF2B contains one gallate and TF3 contains two gallates); inhibition was observed to increase from TF1 to TF3. Control experiments with gallic acid (GA) and propyl gallate (PG) did not lead to any inhibition (Fig. 2a). From these experiments, we conclude that it is the combination of both the theaflavin and gallic acid units that results in DisA inhibition.

**Figure 3:**
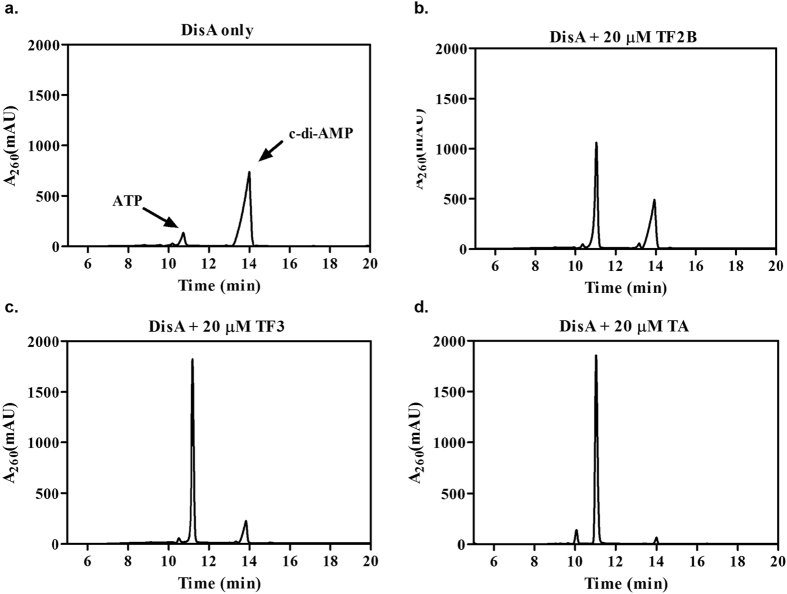
Effect of polyphenols on DisA activity. HPLC analysis of DisA reactions (1 μ M) reactions (**a**) without inhibitor, with (**b**) 20 μ M TA (**c**) 20 μ M TF3 and (**d**) 20 μ M TF2B. The ATP and c-di-AMP peaks are labeled with arrows.

**Figure 4:**
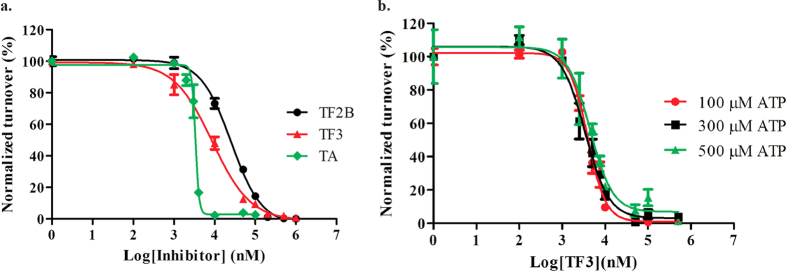
Inhibition of DisA by polyphenol. Half maximum inhibitory concentration, IC_50_ curves of (**a**) polyphenol inhibitors TF2B, TF3 and TA at 1 μ M DisA and (**b**) TF3 at 100 μ M ATP, 300 μ M ATP and 500 μ M ATP with 0.5 μ M DisA. Error bars represent the mean and SEM of triplicate experiments. Curves were generated with GraphPad Prism.

To further explore the inhibition of TA, TF2B and TF3, we first performed HPLC analysis of their respective reactions. Consistent with the results from the coralyne assay, TA was observed to be the most potent of the three; with ~97% inhibition at 20 μ M TA when 1 μ M DisA was used (Fig. 3). TF3 and TF2B followed in that order with ~83% and ~78% inhibition respectively (Fig. 3). We then proceeded to determine the half maximal inhibitory concentration, IC_50_ of TA, TF2B and TF3. Different concentrations of TA, TF2B and TF3 were incubated with 1 μ M DisA, 300 μ M ATP and 11 nM ^32^P-ATP at 30 °C. The amount of c-di-AMP synthesized in the presence or absence of inhibitor was normalized with respect to the amount in the absence of inhibitor and used to estimate the IC_50_ values. A steep dose-response curve was observed when TA (but not TF2B and TF3) was titrated against 1 μ M DisA (Fig. 4a). Steep dose-response curves have been shown to be caused by promiscuous enzyme inhibitors and for such inhibitors, the IC_50_ values have been observed to increase linearly with enzyme concentration^29^. Hence, we determined the IC_50_ of TA at four enzyme concentrations. The IC_50_ values increased from 1.8 μ M at 0.5 μ M DisA to 3.4 μ M at 1 μ M DisA, 16 μ M at 5 μ M DisA and 18 μ M and 10 μ M DisA (Supplementary Fig. S2); consistent with the observations made for non-specific enzyme inhibitors^29^.

The dose response curves for TF2B and TF3 were not as steep as for tannic acid and presumably these two compounds are not promiscuous protein inhibitors. At 1 μ M DisA, the IC_50_ values of TF2B and TF3 were 23.6 μ M and 8.5 μ M respectively, which is lower than what was obtained for the first reported DisA inhibitor, bromophenol thiohydantoin^20^.

### TF3 inhibition does not depend on ATP concentration

Because TA was deemed a promiscuous inhibitor, it was not investigated further. TF3 was selected for further investigation and to gain insight into the type of inhibition exhibited by TF3 (ATP competitive or non-competitive), we determined the IC_50_ of TF3 at various ATP concentrations. Here, we incubated DisA (0.5 μ M) with either 100 μ M ATP, 300 μ M ATP or 500 μ M ATP and increasing concentrations of TF3. IC_50_ values of 3.8 μ M, 3.4 μ M and 4.4 μ M were obtained at 100 μ M ATP, 300 μ M ATP and 500 μ M ATP respectively (Fig. 4b). Since the IC_50_ barely increased upon 3-fold and 5-fold increases of ATP concentration, we conclude that TF3 inhibits DisA in an ATP non-competitive manner.

### TF2B and TF3 are specific inhibitors of DisA

Cyclic di-AMP signaling in *B. subtilis* is regulated by DisA, CdaS and CdaA^4^,^30^, which act as DACs and YybT^31^ (recently renamed as *B. subtilis* GdpP^14^) which is the cognate PDE^31^. To determine whether the inhibitors of DisA identified were specific for the c-di-AMP synthase but not phosphodiesterase, HPLC analyses of YybT hydrolysis of c-di-AMP in the absence or presence of the inhibitors were analyzed. About 50% inhibition of YybT activity was observed when incubated with TA (Supplementary Fig. S3). Furthermore, it has been shown that tannic acid inhibition is abolished in the presence of non-ionic surfactants^32^. When incubated with 0.1% Triton X-100, the inhibition of DisA by TA was completely abolished (Supplementary Fig. S4). TF2B and TF3 did not inhibit YybT (Supplementary Fig. S3), confirming our initial assessment (from the DisA dose-response curves) that TA is a promiscuous inhibitor whereas TF2B and TF3 are not promiscuous.

### Analysis of binding of TF3 to DisA

**Figure 5:**
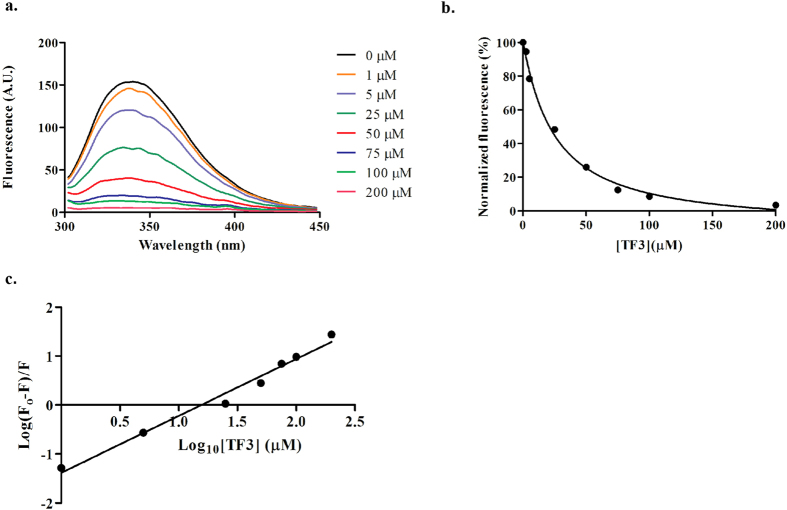
Intrinsic fluorescence analysis of DisA. (**a**) Fluorescence emission trace of DisA (5 μ M) in phosphate buffer (50 mM, pH 7.5) titrated with indicated concentrations of TF3 at room temperature; λ ex =  290 nm and λ em =  300–450 nm (**b**) Plot of normalized fluorescence intensity (at 340 nm) as a function of TF3 concentration. (**c**) The modified Stern-Volmer plot of DisA fluorescence quenching by TF3. F_o_ is the maximum fluorescence intensity in absence of TF3 and F is the fluorescence intensity in presence of TF3. Data points represent the mean and SEM of triplicate measurements plotted using GraphPad Prism.

We determine the binding affinity of TF3 to DisA by measuring the intensity of DisA intrinsic fluorescence when incubated with different concentrations of TF3. When excited with light of wavelength 290 nm, DisA has intrinsic fluorescence with maximum emission at 340 nm. We found that when incubated with TF3, the fluorescence of DisA decreased (Fig. 5a). Assuming a 1:1 binding, we determined the apparent K_d_ of DisA (5 μ M) binding to TF3 using equation (1) 33.


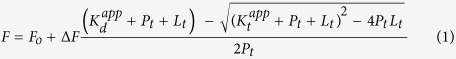


where F and F_o_ are the fluorescence intensity at 340 nm in the presence and absence of ligand respectively, Δ F is the fluorescence change upon ligand binding and P_t_ and L_t_ represent the total protein and concentration. The apparent dissociation constant, 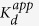
was estimated to be 23 μ M (Fig. 5b).

The decrease in DisA fluorescence upon TF3 binding denote fluorescence quenching. Hence we also used equation (2), the modified form of the Stern-Volmer equation^34^ to determine the binding constant, K_a_ and number of binding sites, n at different inhibitor concentrations, Q (Fig. 5c).


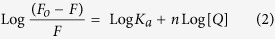


A binding constant, K_a_ of 4.25× 10^4^ *M*^−1^ was obtained and a reciprocal of this gave a dissociation constant, K_d_ of 23.5 μ M. This is in agreement with the apparent K_d_ initially determined from equation (1). The number of binding sites, n on DisA was also estimated to be approximately equal to 1, implying that DisA has a single binding site for TF3. This observation is also in agreement with the earlier 1:1 binding assumption made from equation (1).

## Discussion

C-di-AMP is emerging as a central second messenger that controls various functions in bacteria. Small molecule modulators of c-di-AMP could potentially have applications in medicine, agriculture and synthetic biology. Thus far there is a paucity of small molecules that can be used to “switch” off c-di-AMP synthesis in bacteria and our goal is to identify various small molecules that could be used to inhibit DisA, a c-di-AMP synthase. Polyphenols represent a class of natural products that are primarily known for their antioxidant and antibacterial properties and we were curious to know if these interesting biologically active molecules also inhibit c-di-AMP metabolism enzymes. Indeed there are a few literature reports that suggest that polyphenols inhibit processes in bacteria. Takahashi *et al.*^28^ and Zhao *et al.*^35^ have shown that polyphenols can affect bacterial cell wall. Interestingly some of these tested polyphenols also affect the c-di-AMP synthase, *vide infra.*

Tannic acid (TA), theaflavin-3′ -gallate (TF2B) and theaflavin-3,3′ -digallate (TF3) were found to inhibit the activity of DisA. Of the three, TA was shown to inhibit DisA better than TF3 and TF2B as depicted in Figs 2a and 3. Of note, the inhibition of DisA appeared to depend on the number of gallate moieties on a given aglycone unit. For example, TF3 was more effective than TF2B, which was also more effective than TF1. Also, EGCG was more effective than EGC whilst GC was less effective than GCG and so on. Yet gallic acid on its own or propyl gallate did not inhibit DisA (Fig. 2a), meaning that it is not just the presence of gallate moiety *per se* that caused DisA inhibition but rather the proper spatial presentation of the gallate group that was critical for enzymatic inhibition. It has been reported that gallic acid has antimicrobial activity and it is effective against *S. mutans*, which is one of the causative agents of dental caries^36^. Our data suggests that the effect of gallic acid on bacteria is not via DisA inhibition and is probably via the inhibition of another target; it appears that the gallate moiety is indeed a polypharmacophore unit.

The dose-response curve of TA had a steep slope (Fig. 4a), a signature of a non-specific inhibitor. When tested against YybT, a PDE from *B. subtilis*, TA was found to inhibit the PDE activity of YybT (Supplementary Fig. S3). Others have also reported the inhibition of several enzymes by tannic acid. For example, TA has been shown to inhibit the activity of α -glucosidase, an enzyme targeted in the development of antidiabetic drugs^37^ This inhibition was observed to be stronger than that seen with the antidiabetic drug acarbose^37^. The study also noted the inhibitory effect on trypsin by TA^37^. Yang *et al.* showed TA as a strong inhibitor of epidermal growth factor tyrosine kinase^38^. They also observed that the plant polyphenol inhibited other protein kinases including protein kinase C, mitogen-activated protein kinase and cAMP-dependent protein kinase^39^. The activity of gastric H^+^,K^+^-ATPase^40^ has also been shown to be inhibited by TA. Based on the perceived promiscuity of tannic acid and the fact that it is not drug-like, we decided to focus more on TF2B and TF3, which were observed to specifically inhibit DisA but not YybT (Fig. 3 and Supplementary Fig. 3). The IC_50_ of TF3 against DisA (8.5 μ M) was lower than that of TF2B (23.6 μ M), highlighting the importance of the gallate moiety. Regarding the mode of inhibition, there was no direct correlation between ATP concentration and IC_50_ values TF3 (Fig. 4b), implying that TF3 might be binding to a site on DisA distinct from the nucleotide-binding domain. The binding interaction between DisA and TF3 was found to have an apparent K_d_ of 23 μ M (Fig. 5), which shows moderate affinity.

A number of studies have shown that polyphenols synergize with traditional cell wall-targeting antibiotics and in some cases antibiotic-resistant bacteria could become susceptible to antibiotics via polyphenol potentiation^41^,^42^,^43^. C-di-AMP has been shown to modulate bacterial cell wall synthesis^5^,^8^ so the expectation is that inhibitors of c-di-AMP synthesis could potentiate the effects of cell wall-targeting antibiotics. This work has uncovered a few polyphenols that could be used to modulate c-di-AMP in bacteria but for these molecules or analogs thereof to find practical applications, some limitations need to be addressed. Friedman *et al.* showed that black tea theaflavins possessed antibacterial activity against *B. cereus* at nanomolar levels^44^. However, several other studies have reported rather high minimum inhibitory concentrations for such molecules against other bacteria^41^,^43^. The latter observation might be due to the difficulty of the molecules to enter into bacteria. Use on whole animals might also be limited by rapid metabolism. Perhaps the limitation on membrane permeation could be addressed in the future via acylation of the phenolic moieties. It is likely that the acyl groups would be deprotected by esterases inside cells to uncover the active molecules. Furthermore, the essential gallate moieties are attached to the theaflavin unit via an ester linkage but this type of linkage is unstable towards enzymatic hydrolysis so a more stable stable analog of theaflavin gallates will have to be developed. Beyond the practical application of these molecules to reduce c-di-AMP synthesis in bacteria, the molecules identified in this manuscript could be used to identify the binding site (probably an allosteric site) that would be targeted for the development of potent and specific inhibitors of DisA. Future work, beyond the scope of this paper, will be aimed at gaining structural insights into the binding mode of polyphenols to DisA and could lead to new tactics or design principles to inhibit DisA and ultimately inhibit bacterial cell wall synthesis or other processes that are regulated by DisA.

## Methods

### Protein expression and purification

For protein expression, overnight cultures of *E. coli* containing plasmids of either DisA or YybT were inoculated into 1 L LB medium and cultured at 37 °C. At OD_600_ of 0.6, the cultures were supplemented with 1 mM IPTG to induce expression and incubated at 16 °C with 250 rpm for 18 h. The induced cells were centrifuged at 4 °C and pellets resuspended in lysis buffer [50 mM sodium phosphate buffer, pH 8.0, 300 mM NaCl for DisA and 10 mM Tris-HCl, pH 8.0, 100 mM NaCl for YybT]. The resuspended cells were lysed by sonication and centrifuged at 25,000 rpm for 25 min at 4 °C. The supernatants were passed through HisTrap HP 1 mL columns (GE) and the proteins purified using the Bio-Rad NGC^TM^ Chromatography System at a 1 mL/min flowrate. Elution of proteins was achieved by adding 200 mM imidazole to the lysis buffer. The concentrations of the purified proteins were determined by measuring their A_280_.

### Screening of polyphenols

The polyphenols were screened for DisA inhibition as previously described^20^. Briefly, reactions containing 300 μ M ATP, 10 μ M coralyne, 3 mM KI, 20 μ M inhibitor and 1 μ M DisA in reaction buffer (40 mM Tris-HCl, pH 7.5, 100 mM NaCl and 10 mM MgCl_2_) were set up in triplicates at 30 °C in 96 well plates. A Molecular Devices SpectraMax M5e plate reader was used to measure the fluorescence of coralyne with excitation and emission wavelengths of 420 nm and 475 nm respectively for 30 min with 2 min intervals.

### Enzyme inhibition assays

To analyze the effect of the identified polyphenol inhibitors on DisA, HPLC reactions containing 300 μ M ATP and 1 μ M DisA with or without 20 μ M polyphenol inhibitors were set up at 30 °C. After 30 min the reaction mixture was heated at 95 °C for 5 min and the precipitated proteins were filtered off using a 3 K centrifugal filter (VWR International). Components of the filtrate were then analyzed on a COSMOSIL C18-MS-II Packed column (5 μ m) using 0.1 M TEAA in water and acetonitrile, detecting signals at room temperature with a 260 nm UV detector.

To determine the half maximum inhibitory concentration IC_50_, triplicate reactions containing ATP (at either 100 μ M, 300 μ M or 500 μ M), 11 nM ^32^P-ATP and increasing concentrations of polyphenol inhibitors were mixed in reaction buffer. The reactions were then initiated by adding 1 μ M DisA (or as indicated) at 30 °C for 1 hour. The radioactive components were separated by spotting 0.4 μ L aliquots on TLC plates (EMD Millipore TLC Cellulose). The spots were separated using 1:1.5 (vol/vol) saturated (NH_4_)_2_SO_4_ and 1.5 M KH_2_PO_4_ buffer.

The effect of polyphenol inhibitors on YybT was tested by setting up HPLC reactions containing 50 μ M c-di-AMP and 1 μ M YybT with or without 20 μ M polyphenol inhibitors for 30 min at 37 °C in reaction buffer (100 mM Tris-HCl, pH 8.3, 20 mM KCl, 0.5 mM MnCl_2_ and 1 mM DTT). The reactions were analyzed as described for the DisA reactions.

### Fluorescence spectroscopy and binding characteristics

The binding affinity studies was performed by monitoring the protein fluorescence at 340 nm. Triplicate solutions containing various concentrations of TF3 and DisA (5 μ M) were incubated at room temperature in 50 mM sodium phosphate buffer pH 7.5 for 1 hour. Fluorescence spectra were recorded on a Cary Eclipse Fluorescence Spectrophotometer (Agilent) using a 1.0 cm quartz cell. The excitation wavelength of DisA was 290 nm and emission spectra were collected from 300 nm to 450 nm.

## Additional Information

**How to cite this article**: Opoku-Temeng, C. and Sintim, H. O. Inhibition of cyclic diadenylate cyclase, DisA, by polyphenols. *Sci. Rep.* **6**, 25445; doi: 10.1038/srep25445 (2016).

## Supplementary Material

Supplementary Information

## Figures and Tables

**Figure 1:**
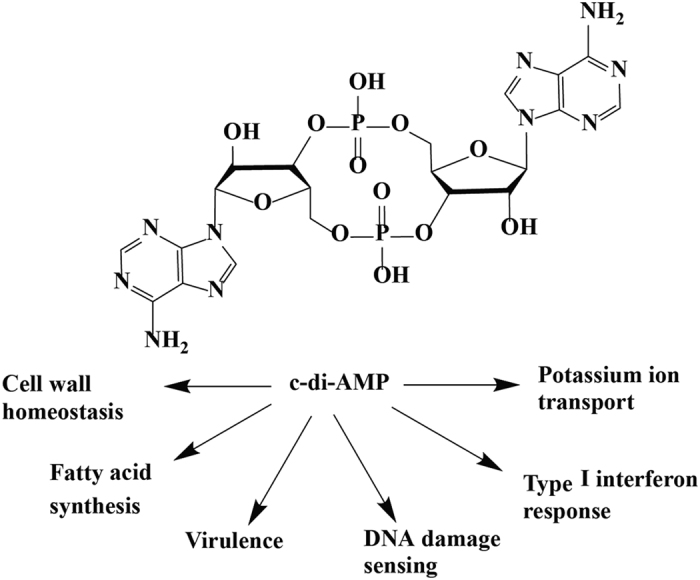
Cellular processes affected by c-di-AMP signaling. Fluctuations in the levels of cellular c-di-AMP cause a myriad of phenotypic changes in different bacteria.
