# Supplementary material for: Inhibition of cyclic diadenylate cyclase, DisA, by polyphenols
Source: Sci Rep. 2016 May 6;6:25445. doi: 10.1038/srep25445 (PMC4858736; doi:10.1038/srep25445)
Supplement: Supplementary Information [file srep25445-s1.pdf]

Supplementary Information

Inhibition of cyclic diadenylate cyclase, DisA, by polyphenols

Clement Opoku-Temeng[a,b,c] and Herman O. Sintim [a,b]*

[a]Department of Chemistry, Purdue University, West Lafayette, IN 47907, USA

[b]Center for Drug Discovery, Purdue University, West Lafayette, IN 47907, USA

[c]Graduate program in Biochemistry, University of Maryland, College Park, Maryland 20742, USA;

*Correspondence author: Tel: +1 (765) 496-6078; Email: hsintim@purdue.edu

ADDITIONAL FIGURES

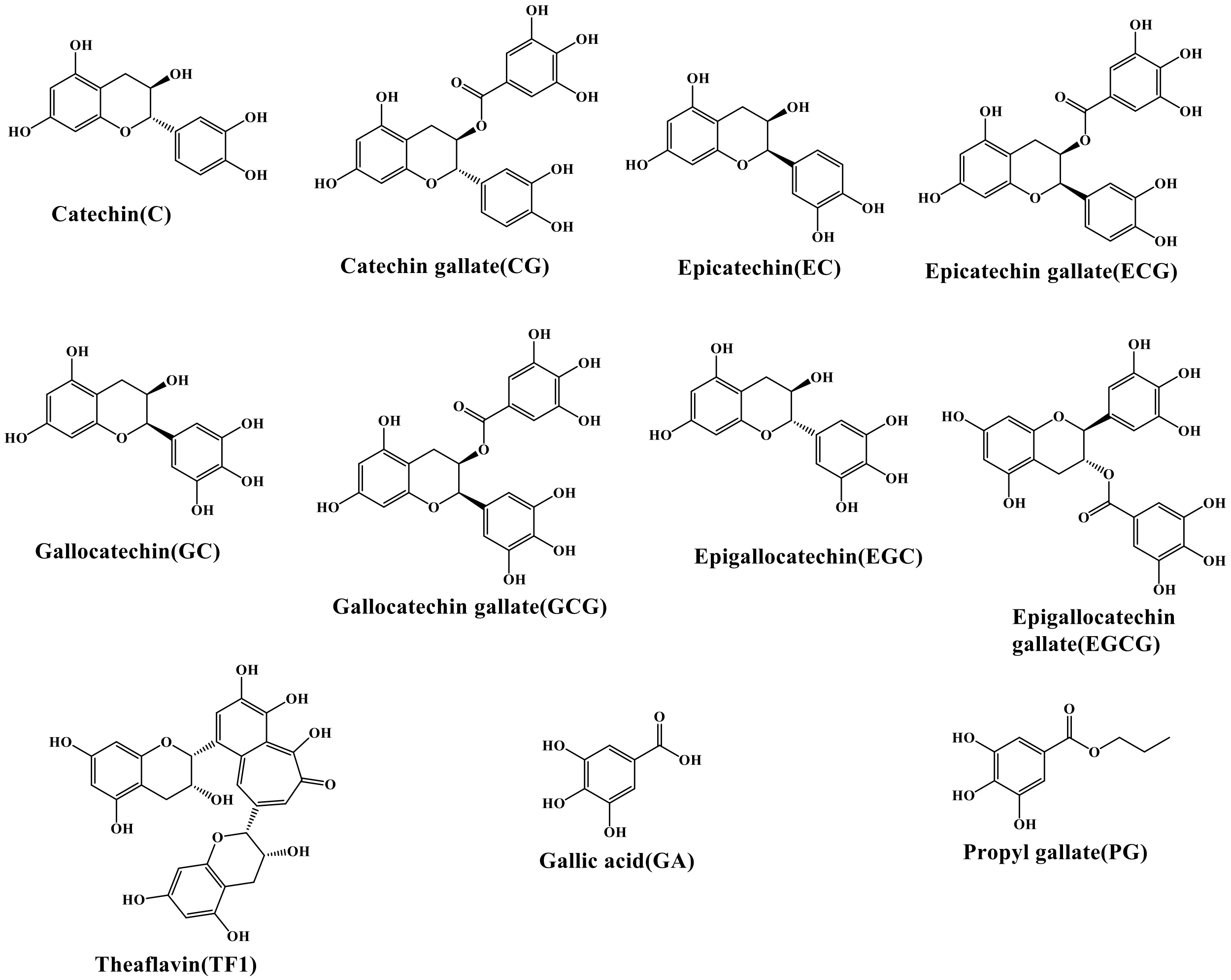

Supplementary Fig. S1 Structures of polyphenols tested against DisA. These polyphenols were tested against DisA and were found to not inhibit c-di-AMP synthesis.

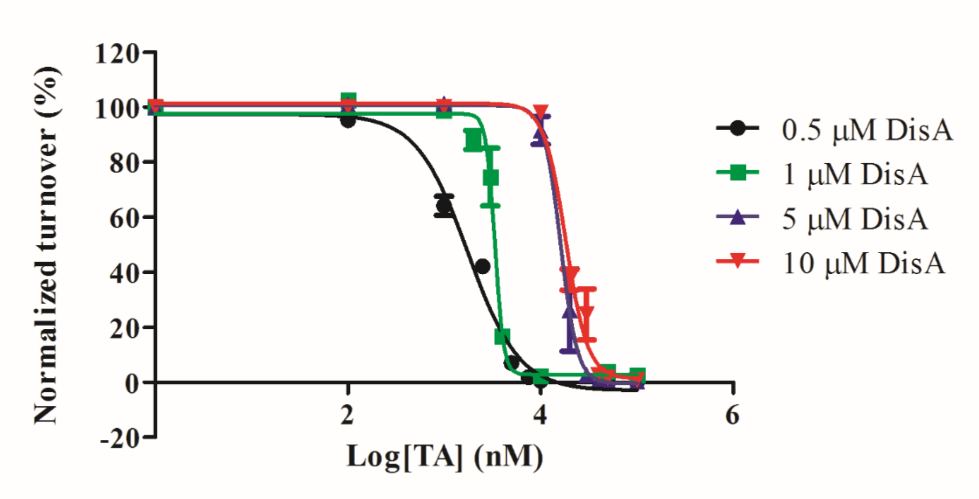

Supplementary Fig. S2 Inhibition of DisA by TA. IC_{50} values of tannic acid were determined at the indicated DisA concentrations. The IC_{50} increased with increasing DisA concentration. Error bars represent SEM of triplicate measurements.

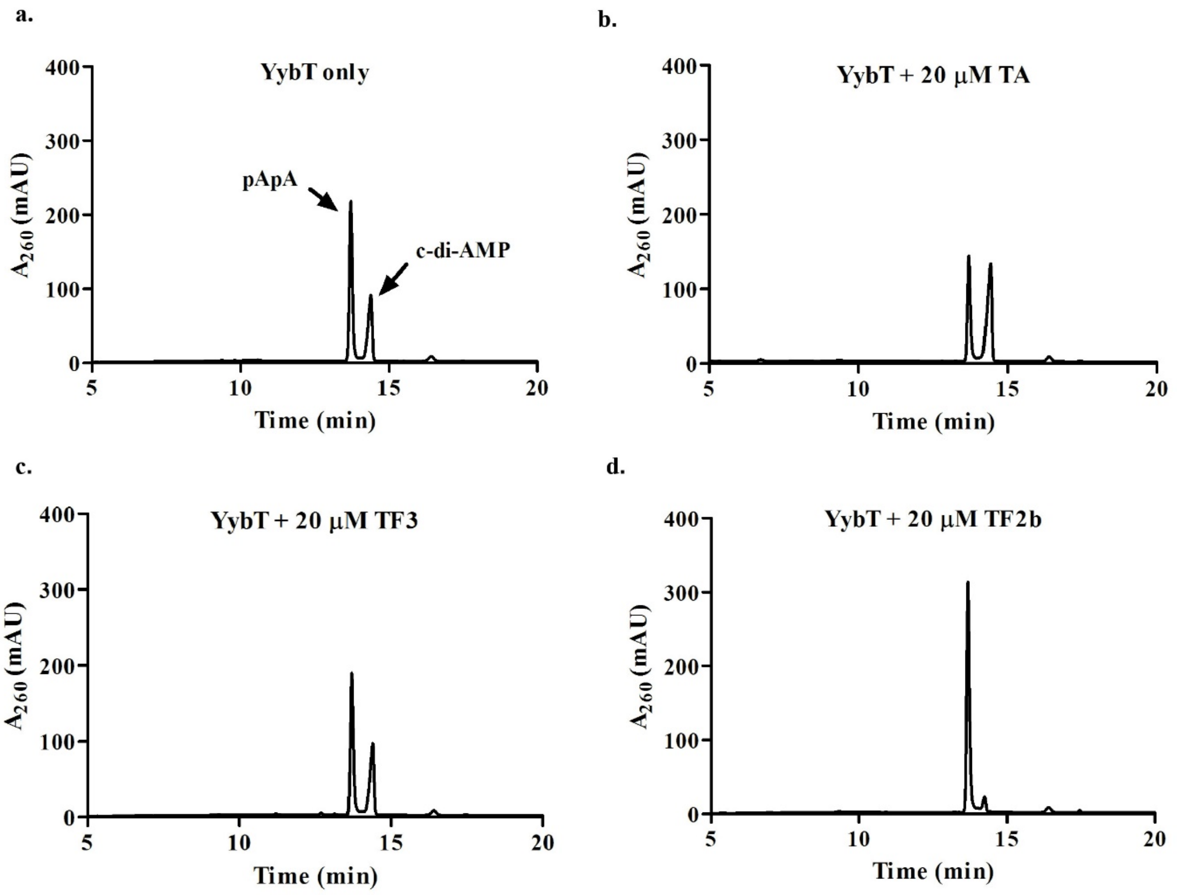

Supplementary Fig. S3 HPLC chromatogram of YybT reactions (A) without inhibitor (B) with 20 μM TA (C) 20 μM TF3 and (D) 20 μM TF2B. The pApA and c-di-AMP peaks are labeled with arrows.

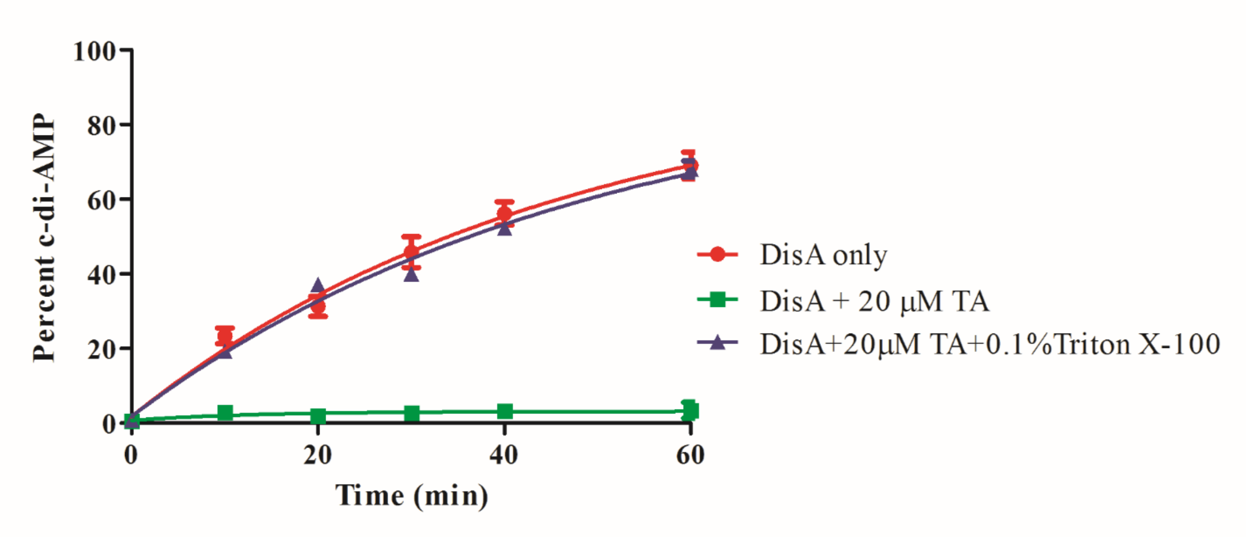

Supplementary Fig. S4 Triton X-100 abolishes TA inhibition of DisA. 20 µM TA completely inhibits the activity of 1 µM DisA. Complete reactivation of DisA was observed at 0.1% Triton X-100. Error bars represent the mean and SEM of triplicate measurements.
